# Abnormal Entropy Modulation of the EEG Signal in Patients With Schizophrenia During the Auditory Paired-Stimulus Paradigm

**DOI:** 10.3389/fninf.2019.00004

**Published:** 2019-02-19

**Authors:** Jie Xiang, Cheng Tian, Yan Niu, Ting Yan, Dandan Li, Rui Cao, Hao Guo, Xiaohong Cui, Huifang Cui, Shuping Tan, Bin Wang

**Affiliations:** ^1^College of Information and Computer, Taiyuan University of Technology, Taiyuan, China; ^2^Translational Medicine Research Center Shanxi Medical University, Taiyuan, China; ^3^Psychiatry Research Center, Beijing Huilongguan Hospital, Peking University, Beijing, China

**Keywords:** schizophrenia, electroencephalogram, fuzzy entropy, sensory gating, complexity

## Abstract

The complexity change in brain activity in schizophrenia is an interesting topic clinically. Schizophrenia patients exhibit abnormal task-related modulation of complexity, following entropy of electroencephalogram (EEG) analysis. However, complexity modulation in schizophrenia patients during the sensory gating (SG) task, remains unknown. In this study, the classical auditory paired-stimulus paradigm was introduced to investigate SG, and EEG data were recorded from 55 normal controls and 61 schizophrenia patients. Fuzzy entropy (FuzzyEn) was used to explore the complexity of brain activity under the conditions of baseline (BL) and the auditory paired-stimulus paradigm (S1 and S2). Generally, schizophrenia patients showed significantly higher FuzzyEn values in the frontal and occipital regions of interest (ROIs). Relative to the BL condition, the normalized values of FuzzyEn of normal controls were decreased greatly in condition S1 and showed less variance in condition S2. Schizophrenia patients showed a smaller decrease in the normalized values in condition S1. Moreover, schizophrenia patients showed significant diminution in the suppression ratios of FuzzyEn, attributed to the higher FuzzyEn values in condition S1. These results suggested that entropy modulation during the process of sensory information and SG was obvious in normal controls and significantly deficient in schizophrenia patients. Additionally, the FuzzyEn values measured in the frontal ROI were positively correlated with positive scores of Positive and Negative Syndrome Scale (PANSS), indicating that frontal entropy was a potential indicator in evaluating the clinical symptoms. However, negative associations were found between the FuzzyEn values of occipital ROIs and general and total scores of PANSS, likely reflecting the compensation effect in visual processing. Thus, our findings provided a deeper understanding of the deficits in sensory information processing and SG, which contribute to cognitive deficits and symptoms in patients with schizophrenia.

## Introduction

The sensory gating (SG) deficit is considered a core deficit among patients with schizophrenia. SG is a normal suppression function of the brain that filters the input of irrelevant information and is believed to be essential to sustain attention in a changing environment and for appropriate responses to afferent stimuli (Braff and Geyer, 1990; Zhu et al., 2013). Schizophrenia patients have demonstrated deficits in SG, which could lead to sensory inundation, excess irrelevant sensory information in the brain, resulting in abnormal information processing, selective attention and cognitive deficits in patients (Adler et al., 2004; Potter et al., 2006; Shaikh et al., 2011; Dalecki et al., 2016; Zhu et al., 2017).

In the auditory modality, SG has usually been studied in a paired-stimulus paradigm: two brief, identical stimuli are presented with a 400 ms stimulus onset asynchrony (Santos et al., 2010; Sánchez-Morla et al., 2013). However, both stimuli elicit a positive potential 50 ms post-stimulus (P50), and the amplitude of potential to the second stimulus is normally attenuated. This phenomenon was considered a measure of input inhibitory, also called P50 suppression. Previous studies have proven that patients with schizophrenia showed a smaller amplitude than normal controls, possibly related to the deficit in SG (Bramon et al., 2004; Chang et al., 2011; Keil et al., 2016). The P50 suppression impairment reported in schizophrenia was thus assumed to reflect an inhibitory input impairment and was argued to be an endophenotype for schizophrenia (Leonard et al., 1996; Korzyukov et al., 2007; Thaker, 2008; Quednow et al., 2012). Recently, our study suggested that schizophrenia patients showed a smaller amplitude to the first stimulus than that of normal controls, which was attributed to the deficit in SG (Zhu et al., 2017). The poor SG in schizophrenia patients was considered to be more related to the diminished processing of S1 than to the deficient gating of S2 (Blumenfeld and Clementz, 2001; Johannesen et al., 2005).

The electroencephalogram (EEG) contains the dynamic properties of brain activity (Acharya et al., 2015). Recently, the dynamic properties were explored by utilizing complexity analyses such as Shannon entropy (ShEn), approximate entropy (ApEn), and Lempel–Ziv complexity (LZC) (Li et al., 2008; Akar et al., 2015; Molina et al., 2017). By analyzing the resting-state EEG signal, schizophrenia patients showed increased complexity, associated with a higher variability or “irregularity” in their brain signals (Akar et al., 2015; Bachiller et al., 2015). Additionally, a difference was found between schizophrenia patients and normal controls in the frontal and temporal regions (Sokunbi et al., 2014). Among complexity analysis approaches, entropy-based algorithms have been useful and robust estimators to evaluate EEG regularity or predictability (Takahashi et al., 2010; Sharma et al., 2015). Entropies measure the probability of a new pattern in a time series; the greater the probability of generating a new pattern is, the greater the complexity of the sequence will be. The entropy with fuzzy structure showed a great performance, including fuzzy entropy (FuzzyEn) (Chen et al., 2007) and Inherent fuzzy entropy (Inherent FuzzyEn) (Cao and Lin, 2018). Moreover, FuzzyEn and Inherent FuzzyEn have been widely applied in the feature extraction and classification of EEG signals in the area of Alzheimer's disease, epilepsy, migraine and healthcare applications (Cao et al., 2015, 2018a,b,c; Xiang et al., 2015).

In addition to analyzing the complexity of the resting state, the complexity of brain activity during information processing was further analyzed. Normal controls displayed decreased complexity (entropy) during tasks (Li et al., 2008; Bachiller et al., 2015; Chu et al., 2017). However, patients showed a significant reduction in task-related changes compared with controls. For example, schizophrenia patients showed a significant reduction in response to both target and distractor tones in an auditory oddball paradigm using spectral entropy (SpEn) (Bachiller et al., 2015). By analyzing the EEG signals evoked by three different types of emotions, the ApEn at the Fz electrode was significantly associated with the total scores of Positive and Negative Syndrome Scale (PANSS) in schizophrenia patients. Furthermore, normal controls and markedly ill schizophrenia patients could be classified with an identification as high as 81.5% (Chu et al., 2017). These entropies and other complex measurements demonstrated that the dynamic properties were sensitive to the neural activity and state of the brain, and provided an important approach to investigate the mechanisms of abnormal cognition in schizophrenia patients. Using the auditory paired-stimulus paradigm, schizophrenia patients showed deficits in sensory information processing and SG, contributing to cognitive deficits and symptoms in these patients (Light et al., 2000; Johannesen et al., 2005; Dalecki et al., 2016; Zhu et al., 2017). However, the mechanisms of entropy modulation during the auditory paired-stimulus paradigm in schizophrenia remain unclear.

In the present study, we used FuzzyEn, a simpler entropy with a fuzzy structure, and an auditory paired-stimulus paradigm to analyze the dynamic complexity of EEG signals, between schizophrenia patients and normal controls, in order to further investigate the entropy modulation mechanisms of SG in schizophrenia. Accordingly, we found abnormal SG in the frontal and occipital regions. FuzzyEn is a useful method to study the complexity of SG and to look for dynamical evidence of abnormal SG in patients. It is also a potential way to study the complexity of the brain diseases.

## Materials and Methods

### Participants

In our study, 61 schizophrenia inpatients [41 men and 20 women, mean age = (37 ± 1.25)] from the Beijing Huilongguan Hospital participated in the experiment. The patients fulfilled the diagnostic criteria for schizophrenia according to the Structured Clinical Interview for the Diagnostic and Statistical Manual of Mental Disorders, Fourth Edition (DSM-IV) (Guze, 2006). The exclusion criteria included cardiovascular or neurological disease, a history of a head injury with loss of consciousness, physical abnormalities, and meeting DSM-IV criteria for substance dependence or current mood or anxiety disorders. They had been treated with the antipsychotic medication of a stable dose for more than 1 month, and did not take clozapine, were pregnant or breastfeeding. The average duration of illness was 14.22 years. The symptomatology was assessed by the PANSS (Kay et al., 1987).

Additionally, 55 age- and sex-matched normal controls were recruited from the staff of the Beijing Huilongguan Hospital [31 men and 24 women, mean age = (41 ± 1.59)]. None of the normal controls had any history of mental illness or substance abuse.

There was no statistically significant difference in age, sex ratio and education between the normal controls and schizophrenia patients. All the participants had normal hearing abilities. The protocol was approved by the ethics committee of the Beijing Huilongguan Hospital, and written informed consent was obtained from all participants after the procedures had been fully explained. The demographical and clinical evaluations of the subjects are listed in Table 1.

**Table 1:** Demographic and clinical variables of the normal controls and schizophrenia patients.

**Characteristics**	**Normal (*n* = 55)**	**Schizophrenia (*n* = 61)**	***t*/χ^2^ value**	***P*-value**
Mean age (SE), year	41.29 (1.58)	37.87 (1.24)	0.464	0.597
Male/Female	31/24	41/20		
Mean illness course (SE), year		14.22 (1.19)		
Mean (SE) age at disease onset		23.89 (0.90)		
**MEAN (SE) PANSS SCORE**				
Positive score		19.63 (0.95)		
Negative score		18.87 (0.93)		
General score		35.12 (1.27)		

*PANSS, positive and negative syndrome scale*.

### Data Recordings

The experiment was implemented in an acoustically and electrically shielded room. All the subjects were asked to wash their hair to ensure clean skin. They were seated in a comfortable chair and were asked to relax and focus their eyes on the “cross” symbol 80 cm ahead. The recording of auditory-evoked potentials was performed using the signal generator and data acquisition system of a fully functional digital 64-channel electroencephalography system (Brain Products, Germany) complying with the international 10–20 system. The resistance of all electrodes was <5 kΩ. EEG was acquired with a sampling frequency of 500 Hz. Eye movements were recorded via electrooculography using Ag/AgCl disc electrodes that were placed at the outer canthus and below the right eye.

**Figure 1:**
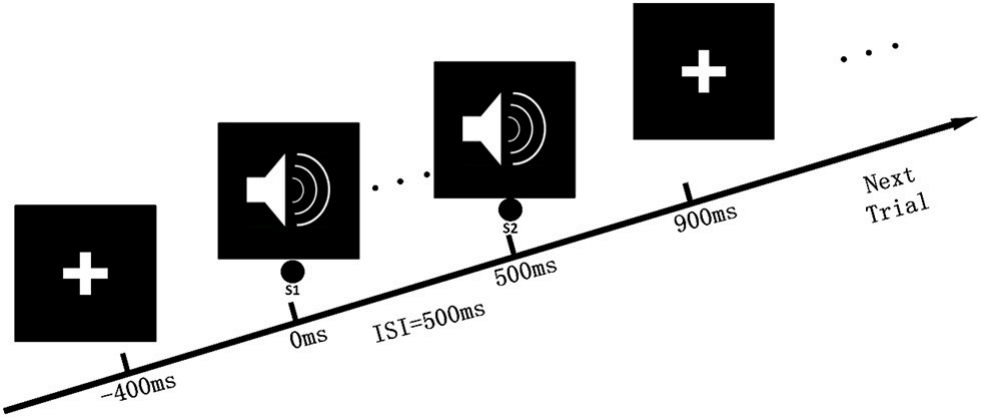
Auditory paired-stimulus paradigm. In the experimental procedure, the subject remained quiet and sat in a chair, facing the “cross” symbol on the front screen, and received paired-clicks through the ears. The time interval between the two stimuli was 500 ms, and 60 repetitions were conducted. Each test interval was 10 s.

The classical auditory paired-stimulus paradigm was used in our previous studies (Tan et al., 2014). The auditory paired-stimulus paradigm (S1 and S2) was also introduced in this study, and 60 paired clicks were delivered binaurally through headphones. The intertrial interval was 10 s with a 500 ms interstimulus interval between S1 and S2. The clicks consisted of broad-band square waves that were 1 ms in duration with an intensity of 80 dB. Electrical signals from the scalp were amplified and bandpass filtered with a 0.01–100-Hz analog filter and without a 50-Hz notch filter. We evaluated SG by computing the complexity of the auditory-evoked response in the auditory paired-stimulus paradigm. The experimental process of data acquisition is shown in Figure 1.

### Data Preprocessing

The whole process of data preprocessing was performed on BrainVision analysis 2.0. First, we chose the average reference electrode that is commonly used in EEG data preprocessing. Next, digital filtering used a bandpass of 0.5~50-Hz and 24 dB/oct. EEG recordings were then segmented into 1,300 ms-length epochs from −400 to 900 ms with respect to the onset of S1 (60 samples per subject). Baseline correction was performed using the averaged EEG data from −400 to 0 ms before the S1. Electrooculogram (EOG) exerted great influence on EEG; thus, ocular correction was necessary to correct the muscle influence caused by eye movement or blinking. The Artifact Rejection transform was asked to search the data set for physical artifacts following segmentation and to remove or mark segments with artifacts. Then, by enabling individual channel mode, “Bad Interval” markers were written in channels in which artifacts occurred. The segments with artifacts were removed and the new data set only contained the segments without artifacts. Next, we exported the EEG data of 60 electrodes for further analysis.

### FuzzyEn Algorithm

Abundant research has indicated that entropy-based approaches have revealed novel insights into various brain activities, in order to understand the temporal dynamics of complexity. FuzzyEn uses a fuzzy membership function to measure the degree of similarity of vectors, rather than the two-valued function in the SampEn-based algorithm, so the calculated entropy values are continuous and smooth (Chen et al., 2007). Additionally, compared with ApEn and sample entropy (SampEn), FuzzyEn has less dependence and sensitivity to phase space dimension and similarity tolerance. The robustness and continuity of measure values are therefore better. The algorithm is described below.

The phase-space reconstruction is performed on *u*(*i*) according to the sequence order, and a set of m-dimensional (*m* ≤ *N* −2) vectors are obtained as follows:

(1) $$\begin{array}{r} S_{i}^{m}=\left\{u\left(i\right),u\left(i+1\right),\ldots ,u\left(i+m-1\right)\right\}-u_{o}\left(i\right) \end{array}$$

Fuzzy membership function as follows:

(2) $$A\left(x\right)=\{\begin{array}{ll} 1, & x=0\\ exp\left[-ln(2){\left(\frac{x}{r}\right)}^{2}\right], & x>0 \end{array}\;$$

where r is the similarity tolerance.

(3) $$\begin{array}{l} d_{ij}^{m}=d\left[s_{i}^{m},s_{j}^{m}\right]=\begin{array}{c} max\\ k\varepsilon \left(0,m-1\right) \end{array}\{|u\left(i+k\right)-u_{o}\left(i\right)\\ \;-\left(u\left(j+k\right)-u_{o}\left(j\right)\right)|\}\\ \left(i,j=1\sim N-m,j\neq i\right) \end{array}$$

$d_{ij}^{m}$, the distance between two vectors $s_{i}^{m}$ and $s_{j}^{m}$, is the maximum difference values between the corresponding elements of the two vectors. According to the fuzzy membership function, the similarity degree $D_{ij}^{m}$ between two vectors $S_{i}^{m}$ and $S_{j}^{m}$ is as follows:

(4) $$\begin{array}{r} D_{ij}^{m}\mu \left(d_{ij}^{m},n,r\right)=exp\left(\frac{-{\left(d_{ij}^{m}\right)}^{n}}{r}\right) \end{array}$$

Defining the function *φ*(*n, r*), and repeating the steps above in the same manner, a set of (m + 1)-dimensional vectors can be reconstructed according to the order of sequence, defined as *φ*^*m*+1^(*n, r*). Finally, the FuzzyEn value for the time series with a sequence length of N can be expressed as follows:

(5) $$\begin{array}{r} FuzzyEn\left(m,n,r,N\right)=ln\varphi^{m}\left(n,r\right)-ln\;\varphi^{m+1}\left(n,r\right) \end{array}$$

Generally, too large of a similarity tolerance will lead to a loss of useful information. However, if the similarity tolerance is underestimated, the sensitivity to noise will be increased significantly. In the present study, *m* = 2 and *r* = 0.25 × *SD*, where *SD* denotes the standard deviation of the time series (Xiang et al., 2015). A large FuzzyEn value indicates a more random time series, whereas a small FuzzyEn value indicates that the time series is regular.

### FuzzyEn Measurement for the Paired-Stimulus Paradigm

**Figure 2:**
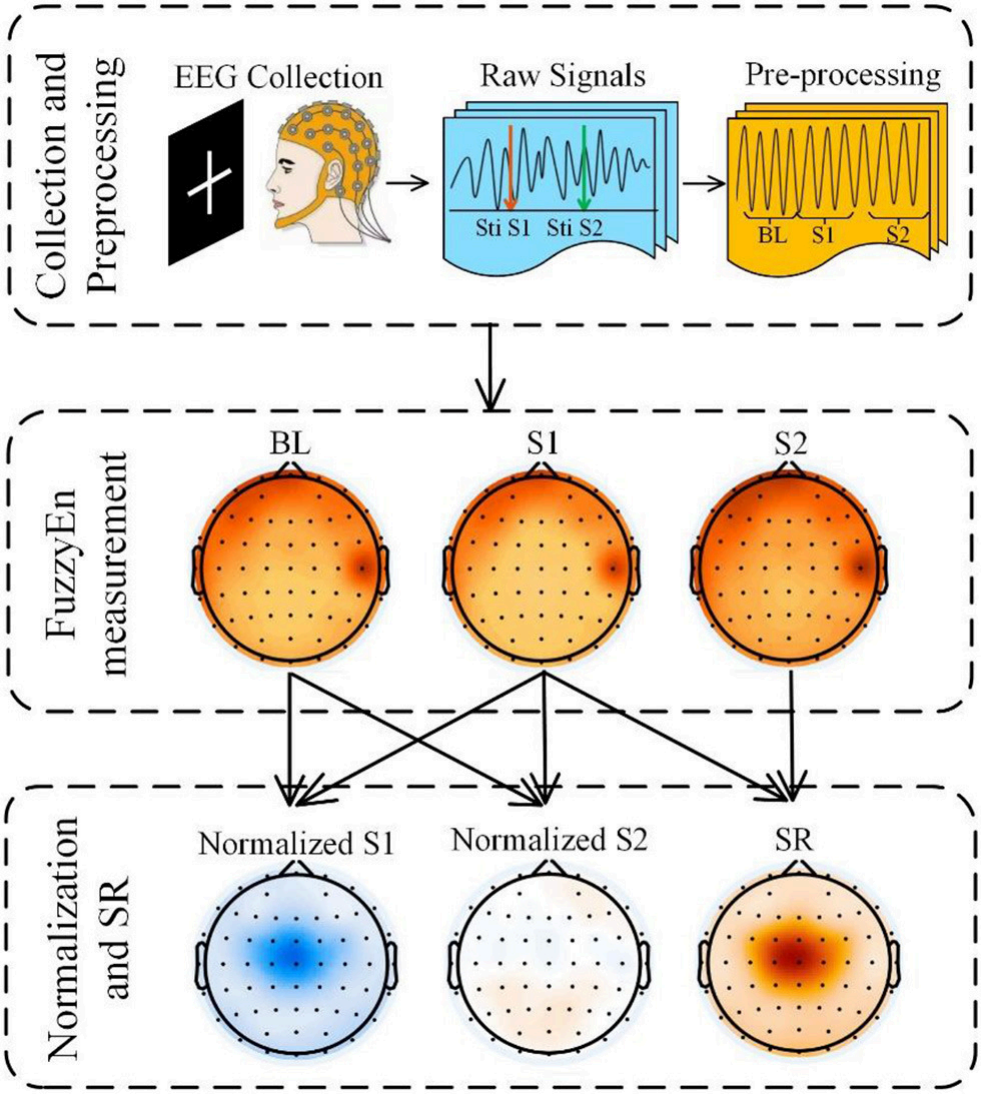
Experimental flowchart. Signal collection and preprocessing, FuzzyEn measurement and Analysis. The EEG signals were recorded using 64 channels of the Vision Recorder system, EEG data pre-processing included re-reference, filtering, segmentation, baseline correction, ocular correction, and artifact rejection; EEG complexity was estimated using FuzzyEn; then normalized values and suppression ratios were calculated.

After preprocessing, we exported the data and calculated the FuzzyEn values using MATLAB 2016b (Figure 2). We determined the entropy values at baseline (BL, range from −400 to 0 ms relative to the onset of S1), S1 (range from 0 to 400 ms), and S2 (range from 500 to 900 ms) for controls and patients. Each channel acquired the corresponding mean value under different conditions for each subject. Finally, we calculated the FuzzyEn mean of each group. To more intuitively observe the changes relative to BL, a normalized method was used, the formula of which is as follows:

(6) $$\begin{array}{r} Normalized\;S=\frac{FuzzyEn\left(S\right)-FuzzyEn\left(BL\right)}{FuzzyEn\left(BL\right)} \end{array}$$

Note: The S indicates the stimulus of S1 or S2.

One indicator of measuring SG is the suppression rate. The traditional method is to calculate the amplitude generated by S1 to suppress the amplitude generated by S2. In our experiment, we analyzed the suppression ratio (SR) from the perspective of complexity as follows:

(7) $$\begin{array}{r} SR=\frac{FuzzyEn\left(S2\right)-FuzzyEn\left(S1\right)}{FuzzyEn\left(S1\right)} \end{array}$$

### Statistical Analysis

Statistical analysis was performed with SPSS 16.0. For the group comparisons of the demographic and clinical variables, we used chi-square tests for categorical variables and independent-sample *t*-tests for continuous variables. To explore differences among conditions, a paired *t*-test was computed. All *p*-values were two-tailed, and the significance level was set to *p* < 0.05 and corrected using the false discovery rate (FDR) (Benjamini and Hochberg, 1995) and Bonferroni correction (Armstrong, 2015). Pearson's r coefficients were computed to investigate the correlations.

## Results

### FuzzyEn Values of EEG During the Auditory Paired-Stimulus Paradigm

**Figure 3:**
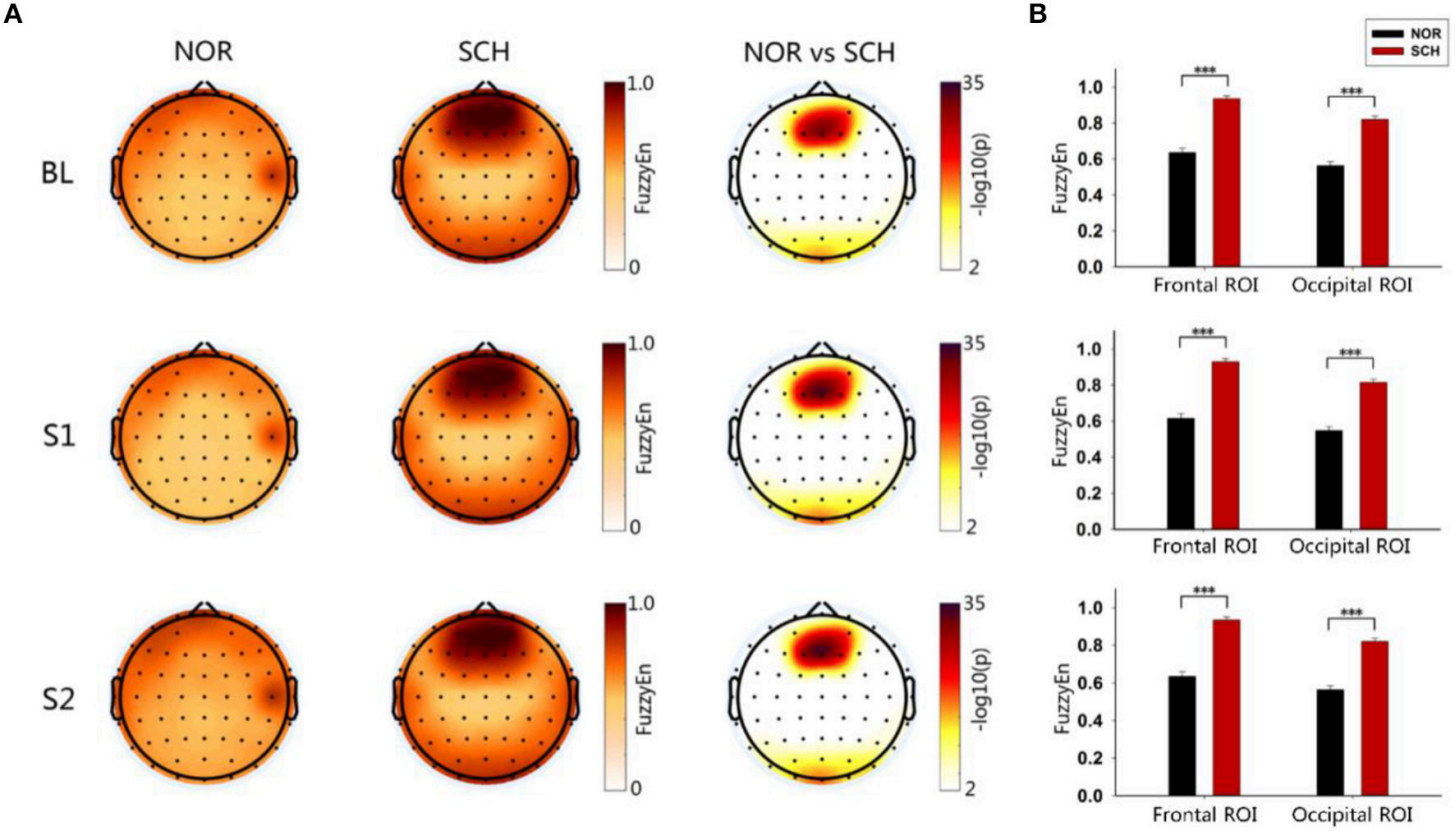
Brain topography and histogram of FuzzyEn values in three states. Panel **(A)** shows the topographic map of entropy in the three states of the two groups. The darker the color in the first two columns was, the greater the entropy would be. The third column took the logarithm of the *p*-value of the statistical test: the darker the color was, the greater the difference would be. Panel **(B)** represents the ROI histograms of the two most significant brain regions. Black represents normal controls, while red represents schizophrenia patients. ***Less than the significance *p*-value of 0.001.

As shown in Figure 3A, FuzzyEn maps of BL, S1, and S2 showed significantly larger FuzzyEn values in schizophrenia patients than in normal controls (*p* < 0.05, corrected) in the frontal and occipital regions. We further defined two regions of interest (ROIs), the frontal ROI (AF3, AF4, F1, F2, Fz), and occipital ROI (Oz, O1, O2), respectively (Figure 3B). By repeated measures analysis of variance (ANOVA), we found a significant main effect of group [*F*_(1, 113)_ > 204.036; *p* < 0.001] and condition [*F*_(2, 226)_ > 14.699; *p* < 0.001] in these two ROIs. There were significant group-by-condition interaction effects on the frontal ROI [*F*_(2, 226)_ = 12.094; *p* < 0.001] and occipital ROI [*F*_(2, 226)_ = 24.297, *p* < 0.001]. By *post hoc* test, the schizophrenia patients showed larger FuzzyEn values than the normal controls in two ROIs, especially in the frontal ROI (*t* > 15.159; *p* < 0.001, corrected). Such differences in the S1 condition were most significant (*t* = 15.845; *p* < 0.001, corrected).

**Figure 4:**
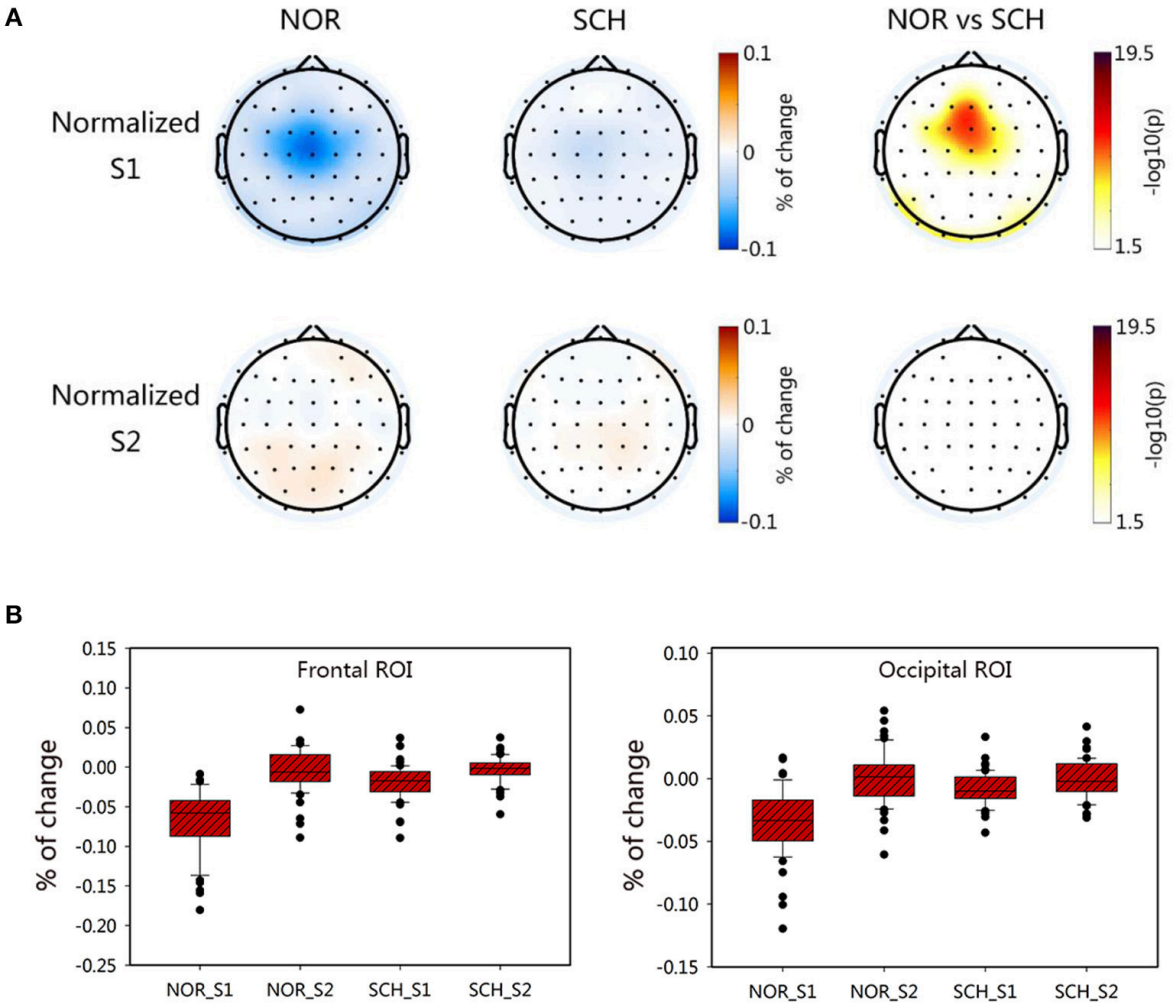
Characteristic distribution of the normalized values for S1 and S2 in each group. Panel **(A)** shows the topographic map of the normalized values of the two groups. The darker the color was in the first two columns, the greater the value would be. The third column took the logarithm of the *p*-value of the statistical test; the darker the color was, the greater the difference between groups would be. Panel **(B)** represents the normalized ROI box plot of the two ROIs.

To exclude the interference of the BL status, we normalized the FuzzyEn values of S1 and S2 relative to that of BL. The brain topographic maps of normalized values are shown in Figure 4A. We found that schizophrenia patients had significantly larger normalized values of S1 than the normal controls in the frontal and occipital ROIs (*p* < 0.05, corrected), values that were in line with the FuzzyEn maps. However, there were no differences in the normalized values of S2 (*p* > 0.2, corrected). We also exported the normalized values in the frontal and occipital ROIs (Figure 4B). Repeated measures ANOVA tests also found significant differences in group [*F*_(1, 113)_ > 9.495; *p* < 0.01] and condition [*F*_(1, 113)_ > 135.079; *p* < 0.01]. Importantly, we found significant group-by-condition interaction effects on the frontal ROI [*F*_(1, 113)_ = 67.597; *p* < 0.001] and occipital ROI [*F*_(1, 113)_ = 59.878; *p* < 0.001]. The *post hoc* test showed significantly larger normalized values in the S1 condition for schizophrenia patients than those for normal controls in these two ROIs (*t* > 8.370; *p* < 0.001, corrected). By contrast, there were no such differences in the S2 condition.

### Complexity Suppression in Sensory Gating

**Figure 5:**
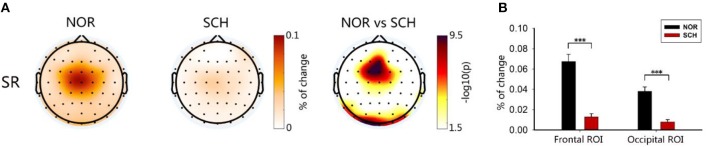
Brain topographic map and ROIs. In **(A)**, the first two plots represent the suppression ratios, and the darker the color was, the greater the suppression ratio would be. The third plot was the result of the two-sample *t*-test. The logarithm was taken, and the darker the color was, the greater the difference would be. Panel **(B)** shows the ROI histogram, including the frontal ROI and occipital ROI. Black and red represent the suppression ratios of the controls and patients, respectively. ***Less than the significance *p*-value of 0.001.

Based on the theory of SG, the strength of SG was determined by calculating the gating ratio. A lower gating ratio indicated weaker SG function. The FuzzyEn map is shown in Figure 5A. We determined two ROIs (Figure 5B), the frontal ROI (FC1, FCz, Fz) and occipital ROI (Oz, O1, O2). In these ROIs, the suppression ratios of normal controls were significantly higher than those of schizophrenia patients [frontal ROI (*t* = 8.578; *p* < 0.001, corrected) and occipital ROI (*t* = 7.862; *p* < 0.001, corrected)].

### Correlation of FuzzyEn Values in the Frontal and Occipital ROIs

**Figure 6:**
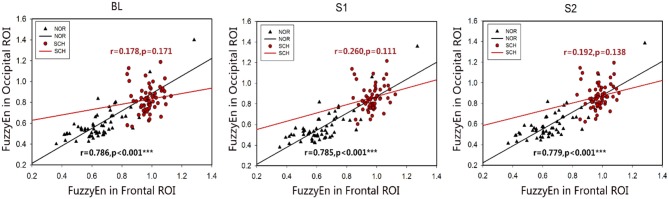
Scatter plots of the associations between the frontal ROI and occipital ROI in normal controls and schizophrenia patients. Black represents normal controls, and red represents schizophrenia patients. The two ROIs in the normal controls showed a positive correlation. No correlation was found between the two ROIs in the patients. ***Less than the significance *p*-value of 0.001.

Figure 6 shows the correlation of FuzzyEn values between the frontal and occipital ROIs. Normal controls showed a positive correlation under all three conditions (*r* > 0.779; *p* < 0.001), while there was no correlation between the two ROIs in patients with schizophrenia (*r* < 0.260; *p* > 0.05). Additionally, no correlation was found in the normalized values and suppression ratios in the frontal and occipital ROIs, for both normal controls and schizophrenia patients.

### Relationships Between Complexity and Clinical Features

The PANSS was mainly used to assess the presence or absence of schizophrenia and the severity thereof (Kay et al., 1987). We performed correlation analyses between the PANSS scores and mean FuzzyEn values of electrodes with a significant difference among the three states (Table 2). The FuzzyEn values showed positive correlations with positive scores of PANSS (PANSSP) in Fz and AF3, as well as with negative scores of PANSS (PANSSN) in AF3 (*r* > 0.260; *p* < 0.05). By contrast, the FuzzyEn values in Oz and O2 (*r* < −0.263; *p* < 0.05) exhibited a negative correlation with the general scores of PANSS (PANSSG) and total scores of PANSS (PANSSTOTAL). No correlation was found between the PANSS scores and normalized values and suppression ratios in patients.

**Table 2:** Results of the correlation analyses between the FuzzyEn and PANSSP, PANSSN, PANSSG, and PANSSTOTAL scores in the patient group.

	**BL (*r, p*)**	**S1 (*r*, *p*)**	**S2 (*r*, *p*)**
**Fz**			
PANSSP	0.279, 0.031*	0.260, 0.044*	0.289, 0.025*
**AF3**			
PANSSP	0.260, 0.045*	0.261, 0.044*	0.266, 0.040*
PANSSN	0.274, 0.034*	0.282, 0.029*	0.271, 0.036*
**Oz**			
PANSSG	−0.294, 0.023*	−0.292, 0.023*	−0.299, 0.020*
PANSSTOTAL	−0.268, 0.039*	−0.263, 0.042*	−0.271, 0.037*
**O2**			
PANSSG	−0.352, 0.006**	−0.356, 0.005**	−0.347, 0.007**
PANSSTOTAL	−0.272, 0.036*	−0.277, 0.032*	−0.269, 0.037*

The first number is the Pearson correlation coefficient, and the second number is the significance.

* *Less than the significance p-value of 0.05*;

** *Less than the significance p-value of 0.01*.

## Discussion

The abnormality of SG is one of the important mechanisms in schizophrenia (Bramon et al., 2004; Chang et al., 2011). In this study, FuzzyEn was used to analyze the dynamic properties of the EEG signal during the paired-stimulus paradigm, to detect evidence of the complexity of abnormal entropy modulation in schizophrenia. We found that schizophrenia patients showed significantly higher FuzzyEn values in the frontal and occipital ROIs. Relative to BL, schizophrenia patients showed a smaller decrease in FuzzyEn values in conditions S1 and S2 than the normal controls. Moreover, schizophrenia patients showed significantly diminished suppression ratios of FuzzyEn, attributed to the higher FuzzyEn values in the S1 condition. The abnormal FuzzyEn values were positively associated with PANSSP measured in the frontal ROI; by contrast, FuzzyEn values were negatively associated with PANSSG and PANSSTOTAL in the occipital ROI.

### Abnormal Entropy in Schizophrenia

EEG signals were complex non-linear dynamic signals (Akar et al., 2015), and it was challenging to accurately extract the EEG signal characteristics. In the present study, we found that schizophrenia patients had obviously higher FuzzyEn values than the normal controls in the BL and stimuli conditions. Consistent with previous studies, patients with schizophrenia had more complex resting-state neural activity and showed increased complexity across the brain using the LZC, compared with the normal controls (Fernández et al., 2011). Additionally, as reported previously using multiscale entropy, schizophrenia patients had a higher complexity than controls (Takahashi et al., 2010). The increase in FuzzyEn values indicates that few neurons participated in the information processing and increase in dynamic complexity, and is linked to an increase in the number of simultaneously active states reflecting the system's degree of freedom (Bob et al., 2007). Furthermore, entropy is an indicator of the probability of generating a new pattern in a time series (Richman and Moorman, 2000; Chen et al., 2007). Compared with the normal controls, the brain activity of patients with schizophrenia was more active and excited, the probability of generating a new pattern of EEG signals was greater, and the complexity of EEG was higher(Zhao et al., 2013). FuzzyEn measurement in EEG signals might be more suitable to capture imperceptible changes in different physiological and cognitive states of the human brain (Hosseini and Naghibisistani, 2011; Mu et al., 2017).

Our results showed significant differences in FuzzyEn values between controls and patients with schizophrenia in the frontal ROI. Previous studies also found a considerable difference in this region (Li et al., 2008; Mathalon and Ford, 2008; Nenadic et al., 2014; Goldstein et al., 2015). The frontal ROI was responsible for memory problems that were associated with the regulation of behavior and cognitive perception (Highley et al., 2001; Yan et al., 2016a). For schizophrenia patients, the impairment of metacognitive function might be mediated by the frontal ROI (Asmal et al., 2016). It was suggested that a disturbance in dopamine signaling to the prefrontal cortex may underlie the abnormalities observed in this region, in social cognition studies of schizophrenia (Sokunbi et al., 2014).

Moreover, a significant difference was also found in the occipital ROI. It is well-known that the occipital ROI is popularly associated with the processing of vision (Yanl and Wu, 2010). Schizophrenia has been associated with an altered structure and function of the occipital cortex (Bjorkquist and Herbener, 2013). Patients with schizophrenia might have deficits in their attention when they were instructed to fixate on the central cross (Dalecki et al., 2016), thereby causing lower entropy values in the occipital ROI. Moreover, there is sufficient evidence to support the notion of a reduction in the overall volume of the occipital ROI in schizophrenia patients (Bilder et al., 1999).

### Abnormal Complexity in the Processing Stimulus in Schizophrenia

In this study, we found that normal controls showed a larger decrease in the FuzzyEn values when processing the S1 stimulus. Such a decrease of complexity in the task state implied a more intense or widespread activation of the cerebral resources, reflecting the activated state of ‘internal concentration' (Li et al., 2008). Similarly, a previous study also reported that the entropy decrease in normal controls could be associated with an irregularity decrease of the EEG signals during the processing of tones (Bachiller et al., 2015). The complexity measured by LZC decreases from the resting state to the tasking state, due to the increase in synchronization during mental activity (Li et al., 2008). Recently, Thilakvathi et al. demonstrated that the complexity of brain activity comes from the neuronal level (Thilakvathi et al., 2017). The higher the complexity is, the more disordered the neuron activity will be. Thus, the decreased complexity in the tasking state might be related to the increase in neuronal activity synchronization.

However, the FuzzyEn values of S1 in schizophrenia patients were significantly higher than those in the normal controls. Compared with the normal controls, after normalization with BL, the schizophrenia patients also showed a smaller decrease in the S1 condition. A weak decrease in complexity in the S1 condition suggested that schizophrenia patients may be less sensitive to novel or relevant sound stimuli and had a lower degree of reactivity, consistent with the result of event-related potential (ERP) in our previous studies (Zhu et al., 2017). It was previously reported that bioelectrical responses to both novelty and relevance, during an auditory oddball task, were attenuated in patients with schizophrenia. The activity modulation was significantly smaller in schizophrenia patients than in the controls, suggesting that the response to both novelty and salience was flattened in schizophrenia patients. Moreover, other results also showed widespread hypoactivation in response to novelty in schizophrenia patients (Laurens et al., 2005).

### Deficit of Sensory Gating in Schizophrenia

In normal controls, the FuzzyEn values of S2 showed a smaller decrease, a finding that was consistent with the FuzzyEn values of S1. The smaller decrease in S2 was considered as suppression of SG (Boutros et al., 1993; Greenwood et al., 2015). Additionally, the phenomenon of suppression was further confirmed by smaller normalized values in the S2 condition and significant suppression ratios of complexity. As mentioned above, the decreased complexity was found in the tasking state. In the S1 condition, the brain's cognitive system considered the stimulus as novel and relevant, the neurons focused on processing this stimulus, and the neuronal activity became orderly; thus, the probability of a new pattern was reduced, and the complexity decreased. In the S2 condition, S1 activates an inhibitory system (Greenwood et al., 2015), suppressing the response to S2 and filtering out the irrelevant information. The suppression marked the order of brain neuron activity decline, and the probability of a new pattern increased. Thus, these results implied that the complexity was sensitive to evaluate SG by calculating the suppression ratio.

We further found that the suppression ratios of complexity in schizophrenia patients were weak and obviously smaller than those in normal controls. The reduced suppression ratios indicated their abnormal SG in the presence of S2 stimuli. A large body of evidence suggests that a significant proportion of patients with schizophrenia had SG impairments (Bramon et al., 2004; Chang et al., 2011). It was theorized that the positive and perhaps negative symptoms of schizophrenia resulted from sensory overload and/or impairments in the response to sensory input within the central nervous system (Keil et al., 2016). However, we found no difference in the normalized values of S2 between schizophrenia patients and the normal controls. The higher FuzzyEn values in the S1 condition were attributed to the abnormal suppression ratios of complexity in schizophrenia patients. In line with our findings, Adler et al demonstrated that patients with schizophrenia had difficulty processing sequentially presented sensory stimuli (Adler et al., 2004). Some studies showed that SG deficits were more related to a diminished response to the S1 condition in schizophrenia patients than to a deficient gating of the response to the S2 condition (Blumenfeld and Clementz, 2001; Johannesen et al., 2005; Zhu et al., 2017). Molina et al. thought the more dynamic (in terms of modulation) regions in the healthy brain showed hampered dynamics in schizophrenia (Molina et al., 2017).

### Relationships Between Complexity and Clinical Features

In the present study, we further found that FuzzyEn values in the frontal ROI (FZ and AF3) showed positive correlations with PANSSP and PANSSN. Higher FuzzyEn values and higher PANSSP or PANSSN scores indicated a more serious disease (Kay et al., 1987; Leucht et al., 2005; Cerquera et al., 2017). These findings indicated that the higher complexity of EEG signals was associated with the more serious clinical symptoms. Consistent with our result, previous studies have also reported that the physiological and cognitive states of the brain could be determined using complexity measurements in EEG signals (Bachiller et al., 2015; Molina et al., 2017). These findings indicate that complexity could be a useful indicator to reveal the physiological states of the brain and clinical characteristics.

Interestingly, the FuzzyEn values in the occipital ROI (Oz and O2) exhibited negative correlations with the PANSSG and PANSSTOTAL. We thought that these negative correlations were associated with the compensation in visual information processing (Bjorkquist and Herbener, 2013). The occipital ROI would show more loading of visual information processing; thus, the activity of the neurons in the occipital ROI became more orderly, and the entropy values would decrease. Additionally, we found correlations between FuzzyEn values in the frontal and occipital ROIs in normal controls (Figure 6). The schizophrenia patients with more serious clinical symptoms required more attention to focus on the central “cross” symbol and more visual information processing (Dalecki et al., 2016), leading to the decrease in FuzzyEn values and lack of consistency of FuzzyEn values across subjects. The negative associations between FuzzyEn values and clinical symptoms in schizophrenia patients reflected a phenomenon of compensation in the occipital ROI to maintain focus on the central “cross” (Yan et al., 2016b).

No correlation was found between the PANSS scores and normalized values and suppression ratios in patients. Moreover, other studies found no relationship between SG deficits and performance on cognitive tests (Fernã et al., 2013; Sánchez-Morla et al., 2013). Our results proved that the SG deficits may be an indicator associated with chronic schizophrenia itself; thus, they were independent of the severity of the disease (Adler et al., 2004; Turetsky et al., 2007).

### Limitations

There were several limitations in our study. First, all patients had chronic schizophrenia and were undergoing long-term treatment with antipsychotics. We could not distinguish whether the difference in the complexity between the controls and patients was affected by antipsychotic treatments. Therefore, the complexity of SG in schizophrenia warrants further investigation in the first episode, with drug-naïve patients and using a longitudinal design. Second, we could not correlate the ERP component of the resting state and SG with the FuzzyEn values; thus, the relationship between complexity and ERP needs further research.

Moreover, since EEG is composed of non-linear signals, EEG complexity is fundamentally mercurial and varying. Intrinsic modes extracted from empirical mode decomposition (EMD) would benefit from eliminating noise/trends in EEG signals (Huang et al., 1998), which can improve EEG complexity evaluation. So Inherent FuzzyEn, which endows fuzzy membership function with EMD by eliminating trend oscillations, has the robustness to noise, non-linear and non-stable signals. It can also operate EEG signals across a range of time scales (Cao et al., 2018c). Previous research has shown that entropies with a fuzzy structure (Inherent FuzzyEn and FuzzyEn) exhibited better performance, and that the performance of Inherent FuzzyEn was the best (Cao and Lin, 2018). We will use the Inherent FuzzyEn to investigate the abnormal SG in patients of schizophrenic under the auditory paired-stimulus paradigm in the future.

## Conclusions

In this study, FuzzyEn was used to extract the non-linear feature of EEG signals under BL and paired stimuli, focused on the changes in the complexity of SG between normal controls and schizophrenia patients. In our study, we found that the FuzzyEn values of schizophrenia patients were higher than those of the controls, in three conditions in the frontal and occipital ROIs. The increase in FuzzyEn values represented an increase in the probability of the time series producing new patterns in the brain. When processing information in the stimulus condition, the complexities were reduced in the normal controls, but few changes occurred in the patients with schizophrenia. From the perspective of complexity, the suppression ratios of SG in the controls were significantly higher than those in patients with schizophrenia. Additionally, the differences in the complexity of SG were mainly due to S1. FuzzyEn offered the evidence of abnormal SG in schizophrenia, and complexity analysis might be an important way to understand SG in future studies. This study could facilitate the diagnostic interpretation of the complexity for schizophrenia conditions.

## Data Availability

The datasets generated for this study are available on request to the corresponding authors.

## Author Contributions

JX and CT completed the study, performed the experiments, and wrote the manuscript. BW and ST provided advice and guidance. YN, TY, DL, RC, HG, XC, and HC revised the manuscript. JX and BW provided the research ideas. ST provided the raw data.

### Conflict of Interest Statement

The authors declare that the research was conducted in the absence of any commercial or financial relationships that could be construed as a potential conflict of interest.
